# Normalizing alternate representations of large sequence variants across multiple bacterial genomes

**DOI:** 10.1186/1471-2105-16-S2-A8

**Published:** 2015-01-28

**Authors:** Alex Salazar, Ashlee Earl, Christopher Desjardins, Thomas Abeel

**Affiliations:** 1 Broad Institute of MIT and Harvard, Cambridge, Massachusetts, USA; 2 University of California, Santa Cruz, California, USA; 3 Delft Bioinformatics Lab, Delft University of Technology, Delft, Netherlands

## Background and description

Variant-focused comparative genomics enables researchers to study the evolution of distinct genetic characteristics in bacterial populations, while avoiding the difficulties of whole-genome assembly and alignment. A major challenge in using this method is that many variant detecting tools are largely limited to predicting single nucleotide variants (SNVs) and small indels. This is a challenge because bacterial organisms do not only possess SNVs but also harbor much larger sequence variants (LSVs), such as large indels and substitutions (>25 nt), when compared to a reference genome. LSVs have been shown to play a role in shaping important biological aspects such as virulence and drug resistance as well as reporting on population structure [1-3]. Recent variant callers, such as Pilon http://www.broadinstitute.org/software/pilon/, can identify LSVs with single nucleotide accuracy in microbial genomes. However, one remaining challenge is that identical LSVs can be represented non-identically by a single variant detecting tool; this generally results from similarity in the flanking sequence of the variant and variability of the read quality and alignment information in that region across the different strains. As a result, alternate representations of large variants make it difficult to perform downstream analyses - such as association studies - that depend on consistent representations of variants.

We present Emu, an algorithm that resolves alternate representations of LSVs by comparing variant calls across genomes.

## Results

To evaluate Emu's ability to resolve alternate representations of LSVs, we introduced 179 simulated LSVs into the H37Rv genome--a carefully curated and finished reference genome for *Mycobacterium tuberculosis *(Mtb). We then used Pilon to identify variants in a set of 146 clinical samples of Mtb that were collected in China using the modified H37Rv genome as a reference [4]. We identified a total of 10,001 unique variant representations. The average number of non-identical representations of each simulated LSV was 56 (in the range of 1 to 145). We then applied Emu to identify the non-identical representations across the genomes of the 146 clinical samples and canonicalize them to a single form. Emu reduced the total number of non-identical representations to 676 LSVs bringing the average number of non-identical representations at each LSV to 4, with 15 LSVs reduced to a single representation and no LSV having more than 25 representations.

We then investigated how Emu's ability to resolve alternate representations might impact association analyses, e.g., associating LSVs with population structure. We ran Pilon again on the set of 161 clinical samples from China, but used the unmodified H37Rv genome. Pilon identified a total of 20,512 distinct LSVs when compared to the unmodified H37Rv genome. By applying Emu, the number of distinct LSVs decreased by almost 50% to 10,936 LSVs. Emu also increased the power of association tests on the LSVs. While we initially identified a total number of 69 LSVs that were significantly associated (p < 0.01) with membership to a specific clade, after processing with Emu that number increased to 94.

## Conclusion

Emu enables comprehensive analysis of LSVs in bacterial genomes by reducing the cross-sample noise that results from per-sample variant calls. By normalizing our variant calls with Emu, we increased our power to utilize LSVs association tests. Pilon and Emu are open source tools that can also be applied to identify and normalize variants in other organisms.
